# Co-design of an intervention to enhance healthcare transition for adolescents and young adults with chronic medical conditions

**DOI:** 10.1038/s44401-025-00041-4

**Published:** 2025-10-01

**Authors:** Ashlie Nobilo, Jordana McLoone, Mary White, Hayley Smithers-Sheedy, Jemma Anderson, Raghu Lingam, Cate Bailey, Claire E. Wakefield, Jourdan Emily Hancock, Michael Hodgins

**Affiliations:** 1 Population Child Health Research Group, School of Clinical Medicine, University of New South Wales, Sydney, NSW Australia; 2 Behavioural Sciences Unit, University of New South Wales, Sydney, NSW Australia; 3 Department of Endocrinology & Diabetes, The Royal Children’s Hospital, Melbourne, Australia; 4 The Cerebral Palsy Alliance Research Institute, The University of Sydney, Sydney, Australia; 5 Department of Adolescent medicine, Women’s and Children’s Hospital, Adelaide, SA Australia; 6 Melbourne Health Economics, Centre for Health Policy, University of Melbourne, Melbourne, Victoria Australia; 7 Division of Quality of Life and Pediatric Palliative Care, Stanford University, School of Medicine, California, USA; 8 Sydney Children’s Hospitals Network, Sydney, NSW Australia; 9 The Royal Children’s Hospital, Melbourne, Victoria Australia; 10 School of Public Health, Faculty of Health and Medical Sciences, The University of Adelaide, Adelaide, South Australia Australia; 11 Children’s Hospital at Westmead Clinical School, The University of Sydney, Sydney, NSW Australia; 12 Murdoch Children’s Research Institute, Melbourne, Victoria Australia; 13 Faculty of Medicine and Health, Specialty of Child and Adolescent Health, University of Sydney, Sydney, NSW Australia; 14 Community Paediatrics Research Group, Faculty of Medicine and Health, The University of Sydney, Sydney, NSW Australia

**Keywords:** Health care, Medical research

## Abstract

Adolescents and young adults (AYAs) with chronic health conditions must transition from paediatric to adult healthcare systems, yet many face significant challenges during this process. This qualitative study explored mechanisms influencing healthcare transition and examined components of a novel intervention—Transition Compass—featuring automated messaging, access to a transition nurse coordinator, and educational modules. Data were collected via individual interviews (*n* = 15), AYA-parent dyadic interviews (*n* = 4), and focus groups (*n* = 15) with AYAs, parents/carers, and healthcare professionals (*n* = 89) across five Australian states. Key factors affecting transition success included AYAs’ evolving autonomy, variable expectations and knowledge across stakeholders, communication gaps, disconnects between healthcare systems, and inconsistencies in care. Participants viewed Transition Compass as a promising strategy to support AYAs through this critical phase.

## Introduction

Over 40% of Australian children are living with at least one chronic medical condition (CMC)^1,2^. Chronic medical conditions persist for one year or more, require ongoing medical intervention, and impair functioning^3^. Advances in medical treatment have significantly increased the survival rates of children with CMCs, leading to a growing population of adolescents and young adults (AYAs) who need to navigate the transition from paediatric to adult healthcare services in order to maintain their health and wellbeing^4,5^.

The transition from paediatric to adult healthcare is a complex, multifaceted process^6^. Transition in the context of our study is understood as both transitional care, referring to the comprehensive preparation and support provided to young people and their families as they move from paediatric to adult health services, and transfer, referring to the discrete event of formally moving care responsibility from paediatric to adult providers^7^. We have conflated these terms within our study because, while conceptually distinct, interventions aimed at improving transition need to consider both the preparation to transfer, and the transfer success itself. Poorly managed transitions can lead to adverse outcomes, including loss to follow-up after transfer from paediatric services^8–10^, increased risk of treatment interruptions^11^, increased reliance on crisis-driven and emergency care^12^, and higher risks of morbidity and mortality^13^. Previously identified barriers to successful transition include inadequate or non-existent policies guiding transition, a lack of resources and training provided to healthcare professionals to manage and support transition, and limited AYA knowledge and capacity to navigate the adult healthcare system^14^. Interventions to support the transition process have the potential to improve AYAs’ satisfaction and independence^15^, self-management behaviours^16,17^, medical adherence^17–20^, illness understanding^16^, quality of life^21^, and engagement with healthcare providers and clinic attendance^18–20,22,23^. However, there remains a scarcity of high-quality studies on this topic^24^, and especially a lack of scalable disease-generic transition intervention development studies Most research on designing and evaluating healthcare transition interventions remains limited to specific medical conditions such as spinal cord injuries^25^, sickle cell disease^26–28^, and Type 1 diabetes^29–31^. Additionally, research on healthcare transition to date, lacks consumer-driven and collaborative efforts across adolescent and adult services^32^. Although studies show that interventions co-designed with AYAs are both feasible and acceptable, the involvement of AYAs with CMCs in co-designing transition interventions to date remains limited^33^. This qualitative study, guided by co-design principles and implementation science, aims to identify the barriers and facilitators of healthcare transition for AYAs, as well as the proposed components for a novel intervention called Transition Compass.

## Results

Our analysis focused on factors guided by the CFIR associated with the outer setting, inner setting, and characteristics of individuals. Understanding these determinants provided insights into the complexities of healthcare transition and served as the foundation for understanding how the proposed Transition Compass intervention could mitigate barriers in the transition process. The findings are presented in two sections: the determinants of transition and the proposed Transition Compass intervention. Key quotes are provided in Tables 1–6. A more comprehensive list of quotes organised by CFIR domains and sub domains is provided as supplementary table 1. Table 2 provides an overview of the demographic characteristics of our sample, including the self-reported conditions of the AYAs who participated in the study as well as departments represented by adult and paediatrician HCP participants highlighting the diversity of experiences represented.

**Table 1:** Demographic characteristics of sample

**AYAs and parents**			
AYA Gender	Female	10	
	Male	5	
	Non-binary	2	
Parent Gender	Female	8	
AYA Age range	18−24 years old		
Self- reported conditions (AYAs and Parents)	Anaphylaxis, Anxiety Disorders Asthma, Attention Deficit Hyperactive Disorder, Autism Spectrum Disorder, Central Hypothyroidism Cerebellar Atrophy, Cerebral Palsy, Chronic Anorexia Nervosa, Chronic Lung Disease, Complex Congenital Heart Condition With Fontan, Crohns Disease, Dermographism, Desmoplakin Cardiomyopathy, Diabetes Type 1, Dysglycaemia, Ectodermal Dysplasia Charcot Marie Tooth Disease Type 3, Eczema, Environmental Allergies, Epilepsy, Exercise-Induced Anaphylaxis, Fibromyalgia/Chronic Pain, Hemifacial Hyperplasia, Hydrocephalus, Hypermobile Ehlers Danlos Syndrome, Hypoglycaemia, Hypothermia Regulation, Idiopathic Intracranial Hypertension, Inflammatory Bowel Disease, Intellectual Disability, Neurogenic Bladder, Oesophogeal Atresia, Pancreatitis, Postural Orthostatic Tachycardia Syndrome, Psoriatic Arthritis, Psychosis, Raynaud’s Syndrome, Rett Syndrome Epilepsy, Rett Syndrome Parkinsonism, Sick Sinus Syndrome, Spinal Cord Lesion From Birth, Tourette Syndrome, Tracheoesophogeal Fistula		
**Adult HCPs**			
Department/ specialty	Renal/Endocrinology	7	
	Gastroenterology	4	
	Neurology	3	
	Not Disclosed	2	
**Paediatric HCPs**			
Department/ specialty	Gastroenterology		6
	Not disclosed		6
	Respiratory medicine		5
	Endocrinology		4
	Rheumatology		3
	Rehabilitation		3
	Paediatrics		3
	Neurology		2
	Adolescent Medicine		1
	Dermatology		1
	Rheumatology		1
	Allied Health		1
	Ophthalmology		1

**Table 2:** Determinants of transition key quotations: The process of healthcare transition

Subthemes	Key quotations
Timing of transition	*“One of the barriers sometimes in the timing is if you’re waiting till someone finishes school to transition them, not knowing where they’re going to be going to uni, where they’re going to be living. And so that often [causes] delays… They’re often sort of into the half of the year post finishing school before they’ve actually either sometimes started the transition process, or at least had a referral sent.” Paediatric HCP 19*
Preparedness for transition	*“I didn’t even realise there was like that sort of transition period into an adult system. I suppose I didn’t really get given much information about it, or even realise, ‘oh, actually, I need to transition from paediatric into the adult system’. So, it was very much just as soon as I turned like 18, it was like, ‘oh, okay, you need to start looking for new immunologists’, and things like that… I think I would have liked to be given more information.” AYA 10*
Transition gaps	*“I certainly think the single biggest issue remains the kind of fall off a cliff transition model that we have where you basically go, paediatric service [here], adult service [here], and there’s that massive chasm in between.” Paediatric HCP 21*
Specialist transition services	*“There are a number of patients that we look after that might not meet the transition team guidelines and yet still would benefit from that expertise in terms of empowering them to navigate, they’re still going to have to navigate the adult world of health care, which is so different from the paediatric world. And although we can use their resources, I think they do miss out from having that expertise.” Paediatric HCP 27*

**Table 3:** Determinants of transition key quotations: Healthcare Provider Factors Impacting Healthcare Transition

Subthemes	Key quotations
Connection between paediatric and adult healthcare providers	*“We’d have clinics where we have the adult endocrinologist and the paediatric endocrinologist turn up, and I stopped being involved in any of those for the reasons that it was dependent on all people being available at exactly the same time on exactly the same day. And invariably, one of those three or four would say, just before, I can’t do Thursdays or thanks for that, can we reschedule to next week” Paediatric HCP 17*
Treatment summaries and information transfer	*“Yes, I think the challenge we have is that we don’t share the same records as the paediatric space. So sometimes, I would like to look back at some of the previous imaging, for example, and I can’t, I don’t have access to it unless we go through that laborious process of getting it. So, the handover is brief. It’s usually a one-page letter.” Adult HCP 03*
Disparities between paediatric and adult care approaches	*“Going from the Children’s [hospital] to the adult care, it’s almost like going from flying business class to economy class. Because it’s unfortunately a reality, you have so many services providing excellent care in the paediatric space. And many critical figures in the paediatric space are then missing in the adult world… [there are] a very limited number of specialists who can address those comorbidities in the adult setting.” Adult HCP 07* *“At the Children’s Hospital, they get all the specialists in the room… everyone just seemed to know what was going on. Whereas in the adults, they kind of expect you to tell your story and then you see someone different every time as well, which makes it really, really difficult.” AYA 14*
General Practitioners (GPs)	*“We were told, on many occasions, get yourself a good GP. Well, that’s easier said than done, you know, the good GPs either don’t take new patients, they’ve retired, or they’re just not out there. And to be fair to the GPs, they haven’t got the expertise, and they haven’t got the time to be dealing with a lot of the issues that our children have got. So, I think that that’s really unfair to put that onus on them, as well.” Parent 03*

**Table 4:** Determinants of transition key quotations: Adolescent and young adult factors impacting transition

Subthemes	Key quotations
Complexity of patient needs and resource challenges	*“One other barrier [is] transitioning patients with conditions who don’t fit into a clear adult specialty, an example being children with complex neuro disability with respiratory issues and finding the right hospital-based service willing to engage with such individuals, otherwise, they end up with the GP.” Paediatric HCP 28* *So yeah, even just the frequency of appointments and also as an adult you often not all the time, but often you do have full time work or other commitments, when as a kid, you know, the school which is important, but it is a little kind of easier to get around. And so I think the frequency of appointments impacts life a lot more.” AYA 06*
Financial implications	*“So, often they’re [AYAs] working multiple jobs and skipping on meals, food quality. And with diabetes, it’s very day-to-day decision-making stuff. So, I think there’s a very direct impact. We have quite a few people worried about paying rent and they can’t afford medications.” Adult HCP 03*
Navigating adult healthcare systems	*“When [daughter] first transitioned out of the Children’s Hospital, she was under nine hospitals to cover everything. And so the lack of communication between departments, so lack of coordination, lack of everything… I pretty much just stopped accessing mainstream medicine and we went a whole lot of alternative routes because the hospital system was an absolute nightmare.” Parent 02*
Knowledge and understanding	*“I’ve had some really poor experiences with registrars [in adult services] just like, clearly not understanding the depth of my disability and the different variables that can be just from one person with that disability to another person with the same disability the fact that like, they’re actually we’re not the same person, we won’t have the same health outcomes.” AYA 14*
Expectations and responsibilities during transition	*“I think often people see young people coming into an adult service and assume that they’re fully formed adults and the understanding that adolescence continues well into our 30* *s…, is relatively new thinking and a lot of our senior non-paediatric physicians get frustrated with the ongoing learning that our young people are doing at that time in their life. And they judge it rather than try and meet them where they are.” Paediatric HCP 15*
Self-management and transition readiness	*“They sort of separated myself, from my parents… and I understand why they tried to do by that, because they want you to do it on your own. But I feel like it had the opposite effect in some way… you’re going get a more honest answer if I’m with my parents, because I may say something, but then I don’t articulate how much of an impact it has, where my parents were there and goes, no, that really bothered you” AYA 01*
Complexity of life and competing demands	*“They’re [AYAs] trying to get their independence and break away from their parents in a nice way. But they’re also having the issues on top of all their young adult issues, like their mental health. And a lot of them are wanting to start a new career, education, on top of their disability, so and then accommodation, they’re probably looking at moving out of home. So, they’ve got a lot of issues as well, that need to be looked at and I think, you know, the transition team should be looking at that as a holistic approach as well.” Parent 03*

**Table 5:** Proposed intervention key quotations: Automated messaging intervention and associated transition coordinator

Subthemes	Key quotations
Multi-modal communication and customisation	*“I do know a lot of people really hate phone calls. So that won’t be helpful for everyone, just as long as there’s an option to do that, or option to explain, how it works, and very like, chill, relaxed, language.” AYA 01*
Relatable tone and informal communication	*“I think it’s also important to kind of watch the tone as well, because I think as I do kind of have a bit more hesitant to click on a notification. If I sense the tone is a bit more serious, or it’s a bit more cold, I say. Not really aggressive. But yeah, kind of like really upfront. I do kind of tend to avoid that just personally.” AYA 03*
Human connection and trustworthiness in communication	*“Definitely, having met the person [transition nurse coordinator] would make a difference. Because otherwise it would be like, some robot messaging.” AYA 11*
Adaptability and accessibility	*“I think sometimes my answer might fall in between the cracks. So, yeah, I think that might also be a reason why I’m not going answer in the first place.” AYA 03*

**Table 6:** Proposed intervention key quotations: Education modules

Subthemes	Key quotations
Engagement and attention grabbing	*“I think tone is your most important thing. And then the rest of it will come with it. Because the vaping one, I’m just like, it’s not the way you would film the video with the content you’re thinking of, but it was very sharp, quick and powerful. And it kept you engaged.” AYA 01*
Social media platforms	*“I think TikTok is like a great place for relatability. But I also think that TikTok is a huge rabbit hole of misinformation. I actually worry about like possible embarrassment, like, if someone’s with their friends on TikTok, and this suddenly comes up, they might just feel a bit like, I didn’t want my friends to see that.” AYA 12*
Innovation point of difference	*“We’ve got some [educational resources] already. So, we have an education video for teenagers, it has teen actors in it, it was researched based on all of the safety messages and education they require before they leave… So, you wouldn’t have to reinvent the wheel on those resources, they already are available.” Paediatric HCP 05*
Authenticity and relatability	*“I think it’s important to have a young person that like, I think we all know that it’s just a script but there’s like some subconscious comfort of it’s not coming from a professional” AYA 12*
Educational content	*“I think general health literacy, like, what is Medicare and how to use it and why is that important? What’s the difference between public and private health? And what are your rights as a young person in the health care system? So, actually increasing the ability to advocate for themselves through understanding what they’re entitled to and what their rights are.” Paediatric HCP 15*

We used a process mapping approach to codify the transition pathways across various paediatric healthcare departments, highlighting the complexity and variability resulting from the absence of standardised and consistent processes across locations, institutions, and between diseases (supplementary table 2). This, in addition to our qualitative analysis, helped us to identify factors determining the potential success of interventions supporting transition. Our themes are organised according to the process of healthcare transition, healthcare provider factors impacting transition, and AYA factors impacting healthcare transition.

### The Process of Healthcare Transition

Participants reported that the timing of healthcare transition is variable across institutions, contributing to several challenges and inconsistencies in the care AYAs receive. They shared that some healthcare providers begin the transition process as early as age 15 or 16, while others wait until the AYA is 18 or older. They noted that transition timing can also be influenced by major life changes, such as moving and/or starting university, acknowledging that if the AYA is uncertain about their future plans and location, the ability of paediatric HCPs to refer them to appropriate adult HCPs becomes complicated.

There were mixed views on initiating transition conversations early. While many considered early discussions helpful, some families and AYAs were not ready to fully engage with the information. Despite this, there was a generally strong desire for advanced notice about the differences between paediatric and adult care, to facilitate better preparation and avoid AYAs and families being unaware of the transition until it was imminent. Participants reported that a lack of AYA preparedness led to confusion and gaps in care, as adult services were less structured and frequent, compared to the regular, coordinated appointments in paediatrics. Additionally, participants reported that paediatric HCPs sometimes lacked prior awareness that an AYA was due for transition, only recognising the need at what should be their final paediatric appointment. This lack of early awareness hindered their ability to adequately prepare AYAs and their families for transition. Suggestions for improvement included starting discussions by at least around age 16 or 17, potentially earlier for those living with multiple complex and chronic health conditions and/or disability, and providing information and resources to help AYAs understand and adapt to adult care. Additionally, participants noted that offering tours or programmes to introduce young people to adult healthcare facilities could reduce the intimidation and anxiety associated with the transition.

Frequently noted by participants was the gap between AYAs having their last appointment in paediatric services and their first appointment in adult services. Many described this gap as a ‘falling off a cliff’ model of transition and questioned who was responsible for providing care during this period. Many paediatric HCPs described offering interim care on an ad-hoc basis due to long waitlists and delays in the adult system. This gap was compounded by a lack of formal processes to inform paediatric HCP referrers that the AYA had been seen by adult services. This ‘no man’s land’ during the transition left AYAs vulnerable to falling through the cracks, being removed from waitlists, or otherwise unable to progress their care in adult services. AYAs reported gaps in care due to their age, as they were considered too old for paediatric services but too young and/or physically too small for adult equipment and services. Fragmentation was further exacerbated by multi-morbidity, as the timing of transfer to each adult specialist varied. Differences in team acceptance ages led to some aspects of care remaining under paediatric services while others were transferred to adult services.

Participants shared differing opinions on the effectiveness of specialist transition services, which are usually services embedded in paediatric hospitals that support young people transition to the adult health care system and are staffed by medical, nursing and/or allied health professionals. Paediatric HCPs often referred AYAs with complex healthcare needs, such as those requiring multiple specialists, to these services. However, their experiences with the usefulness of these services varied. Additionally, many AYAs who could benefit from specialist transition expertise did not meet the criteria and were therefore excluded from receiving this support.

### Healthcare Provider Factors Impacting Healthcare Transition

Our process mapping activity (supplementary table 2) and interview data indicated transition success was often better supported when there were established relationships between paediatric and adult specialists. Some paediatric HCPs had well established relationships with adult HCPs, however some expressed a lack of awareness and/or availability of relevant adult HCPs, as well as no established processes to acquire this knowledge. Without knowledge of available and appropriate adult HCPs for referrals, paediatric HCPs found it challenging to effectively connect AYAs to suitable adult care. Given the rarity of many paediatric conditions, they are often treated at a central tertiary centre. However, when transitioned to adult care, participants reported that AYAs become dispersed/treated at local hospitals, and paediatric HCPs struggle to maintain networks across all adult teams statewide to facilitate appropriate local referral. HCPs described efforts to navigate these challenges, including establishing joint paediatric-adult clinics, clinicians with dual employment across the paediatric and adult hospitals, or paediatric and adult services offering a period of collaborative, overlapping care. However, these opportunities were often ad-hoc, driven by individual clinician initiatives and hindered by barriers such as lack of administrative support and clinic facilities, difficulty scheduling HCP diaries, cost, and lack of interest or time by some adult HCPs.

AYA participants raised concerns about the transfer of medical information during the transition process. Paediatric records were commonly maintained on different electronic systems than those used in adult settings, with adult HCPs unable to access them. Healthcare professionals frequently relied on brief handovers that lacked essential details such as previous imaging or comprehensive medical histories. This sometimes led to unnecessary repetition of medical investigations. Paediatric HCPs described varying practices regarding the completion of treatment summaries for transfer to adult HCPs. While some paediatric clinicians routinely prepared these summaries, others were inconsistent due to challenges such as lack of formalised processes, time constraints, or difficulty in summarising extensive care spanning up to 18 years. Some HCP participants highlighted the importance of better communication and documentation systems. They proposed solutions such as transition passports or comprehensive transition letters containing vital medical summaries and recommendations for future care, including the current and anticipated frequency of appointments. A facilitating factor for information transfer was having electronic medical systems that were accessible across both paediatric and adult services. Adult HCPs expressed a desire for information on the social supports of the AYA to understanding the best approach to their care.

One significant challenge AYAs and their families faced during healthcare transition was adjusting to the differences in the care environment and the nature of support provided. Participants highlighted a contrast in the level of personalisation and nurturing between paediatric and adult settings. Participants characterised paediatric care as having a comprehensive, wrap-around approach with a strong emphasis on personalised, centralised care, and multi-disciplinary team collaboration. Paediatric services were also commended for providing follow-up and support, particularly around missed appointments, aiming to streamline care through coordinated scheduling. AYAs described a shift from coordinated, multidisciplinary care in paediatrics to fragmented services in adulthood, where accessing support and specialist services became more difficult and bureaucratic. AYAs expressed feeling like “just another number” in the adult system rather than an individual with specific needs. Additionally, adult services did not follow up missed appointments, discharging patients who did not attend or reschedule. In such situations, AYAs would present to care through the emergency department (ED).

Participants highlighted numerous challenges related to the role and expectations of GPs in the transition process. With no direct equivalent to a paediatrician in adult healthcare, participants noted that the GP often becomes the mainstay for AYAs, who many acknowledged are not resourced appropriately to provide this care. Participants noted that many AYAs may not have a regular GP or an established relationship with a GP due to the predominant involvement of paediatricians in their earlier care. The variability in the level of experience, knowledge and availability of GPs exacerbated the issue, with some regions, particularly rural and remote areas, facing severe shortages and others dealing with GPs who lacked the knowledge, confidence, and experience to manage rare conditions, comorbidities, and other complexities associated with CMCs. Participants noted that continuity of care may be disrupted when patients see different GPs at a clinic, rather than a consistent GP. Furthermore, the limited time available in GP appointments was often described as insufficient to address the complexities of chronic conditions.

### Adolescent and young adult factors impacting healthcare transition

Participants reported that AYAs living with complex medical conditions and/or experiencing psychosocial complexity found healthcare transition particularly challenging. This was compounded by a lack of sufficient resources, such as for AYAs living with neurodiversity, mental health conditions and/or experiencing mental ill-health. According to participants, access was particularly challenging for AYAs who did not meet criteria for disability funding support from the Australian government. Geographic disparities further complicated access to adult healthcare, especially in rural and remote areas that lack specialist services, necessitating long travel distances for essential treatments. AYAs reported that accessing mental health services can be particularly challenging, worsened by a shortage of psychiatrists skilled in managing cases involving complex comorbidities. Participants noted that adult services frequently lack the capacity or expertise to address the multifaceted needs of these patients, leaving under-resourced GPs to fill the gap, often inadequately. As a result, many AYAs experienced discontinuity in care, insufficient support, and an increased risk of being lost to follow-up during transition.

Financial difficulties significantly impacted AYAs during healthcare transition. According to HCPs, AYAs struggle to afford adult care including expenses for hospital visits, medications, and specialist appointments. As they “aged out” of coverage under their parents’ insurance, AYAs reported additional expenses which they became responsible for. Medications that were previously low-cost or free in paediatric settings became expensive in adult services, adding to the financial strain. HCPs noted that publicly funded services, particularly psychiatry, were less accessible in adult care, leading some families to pursue costly private specialists to avoid long waiting lists and gaps in care. Travel expenses to appointments and potential income loss from taking time off work further added to AYAs’ financial challenges. AYAs reported needing to work multiple jobs and make compromises in their healthcare decisions to cope with these financial constraints. Balancing new responsibilities such as rent and bills in some instances exacerbated their reliance on parents and/or carers, impacting their independence.

Navigating the adult healthcare system posed significant challenges for AYAs and their families. Families reported struggles with unresponsive specialists, lack of communication between departments, and the need to frequently turn to private health care due to limited public options. This disjointed approach contributed to medical burnout and frustration, as parents and AYAs were required to take on the complex task of coordinating care without the comprehensive support they previously received.

Individuals’ understanding of both the transition process and medical conditions were identified by participants as factors that influenced the transition process. Adult HCPs highlighted that AYAs, paediatric HCPs, and often even adult HCPs may not always be aware of the specific need for different specialties of care. While some AYAs reported that they felt well-informed about their conditions and medical terminology, others lacked this knowledge, contributing to a reduced understanding of the importance of specialised care. Since adult HCPs generally have more experience with adults and an aging population, participants noted that they may lack knowledge about conditions with paediatric onset, especially for rare diagnoses. Some participants reported that HCPs may be unaware of the unique needs of AYAs who are still learning about their health and navigating the healthcare system. Participants reported adult HCPs as lacking empathy for the anxiety or fear AYAs experience when transitioning their health care treatment and management to the adult system.

Participants reported that healthcare transition is often marked by differing expectations and responsibilities between AYAs and HCPs. This project found that a disconnect regularly exists between adult HCPs’ expectations of an AYAs’ readiness for independence and the AYAs’ capacity and preparedness for managing their healthcare needs. AYAs noted that adult HCPs may assume AYAs are fully capable of self-management, expecting them to handle appointments, medication adherence, and health monitoring on their own. AYAs inability to handle the responsibilities had the potential to lead to misunderstandings and inadequate support, where missed appointments or non-compliance are misinterpreted as disengagement rather than a need for further guidance.

AYAs’ self-management skills and transition readiness were identified as factors that may influence successful healthcare transition. Many AYAs acknowledged the importance of increasing independence in managing their healthcare. However, there was a delicate balance regarding parental and/or carer involvement and concerns that their presence might undermine AYAs perceived independence. Some AYAs preferred and felt confident to manage their care independently, while others valued their parents’ continued involvement as a support system. HCPs also recognised the need to encourage self-management and independence, acknowledging that the appropriate level of parental and/or carer involvement is likely to be unique for each AYA and may require a flexible approach. AYAs referred to strategies that enabled them to become more independent in managing their care, such as keeping folders of important medical information to take to each appointment, taking notes during appointments, and using calendars or reminder systems for appointments.

Many AYAs reported experiencing significant life changes contemporaneous to healthcare transition, such as leaving home, attending university, or beginning a job. They reported that having to negotiate competing responsibilities posed challenges in managing frequent medical appointments, which could potentially lead to gaps in healthcare continuity. The need to negotiate and navigate these new responsibilities such as using sick leave or arranging time off work for appointments was described as further complicating their ability to maintain a consistent appointment schedule. Both HCPs and parents recognised the increased risk of AYAs falling through the cracks due to reduced parental oversight, the demands of new responsibilities, and the impact of mental health issues.

### Proposed transition compass intervention

**Fig. 1:**
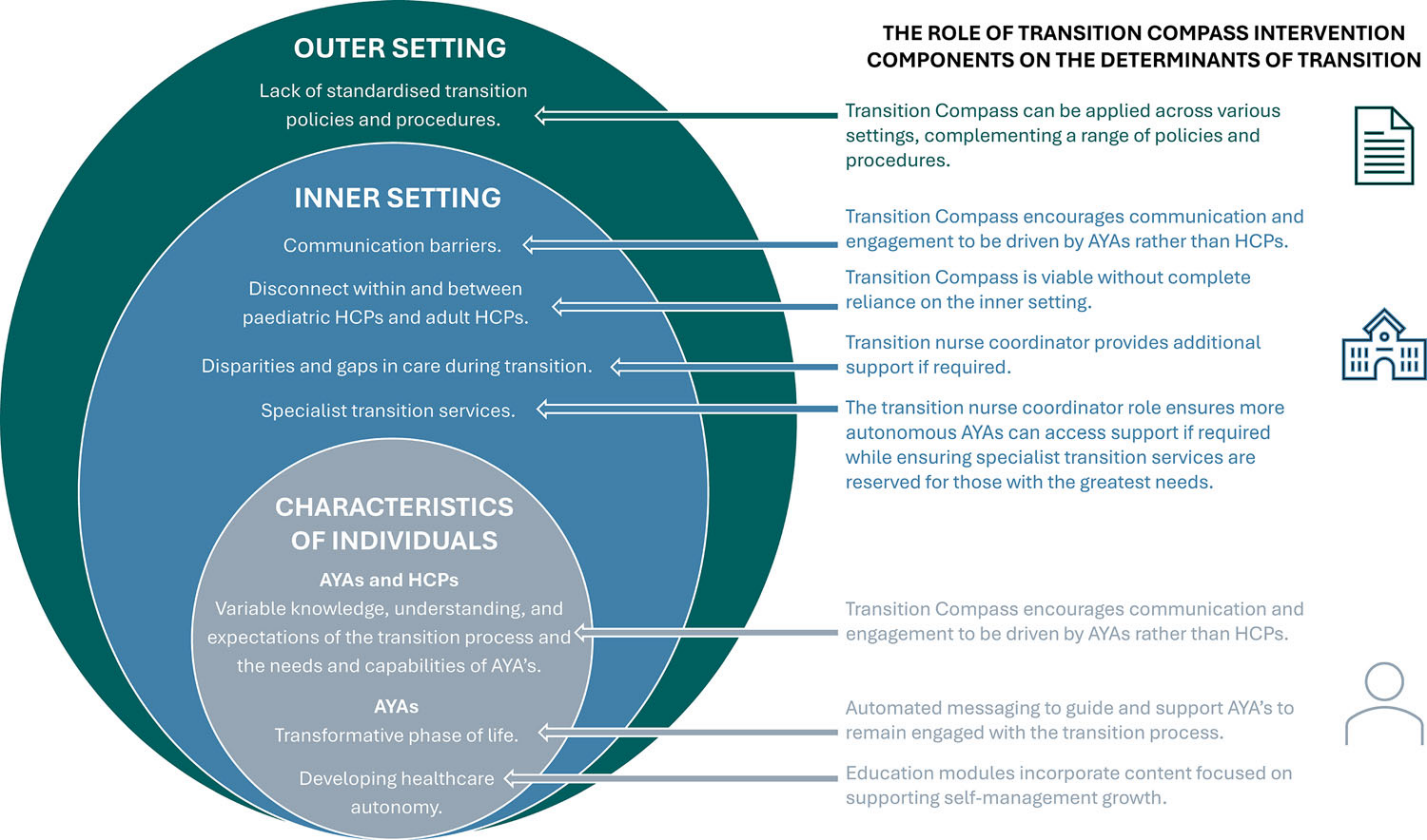
Key determinants influencing the healthcare transition process: Individual, inner setting, and outer setting CFIR domains and the corresponding role of Transition Compass intervention components.

Our findings highlight that healthcare transition is multifaceted and presents a major challenge due to the involvement of two very different healthcare systems. Developing a straightforward solution across both paediatric and adult healthcare settings is unrealistic given the system complexity. Instead of attempting to completely overhaul one or both systems, the determinants of transition identified in this study suggested that an intervention focused on developing self-management skills for AYAs could be an effective and feasible approach (Fig. 1). This approach would empower AYAs to become more independent in managing their healthcare needs and increase their confidence in navigating the intricacies of the healthcare system. We aimed to develop an intervention that could be applied universally, ensuring that AYAs who are independent in managing their own care have access to support if needed, whilst being able to identify those at risk of disengaging from care and directing them to more intensive assistance as needed. We explored participants’ views on the proposed components of the Transition Compass intervention including automated messaging support, connection to a transition coordinator, and education modules.

### Automated messaging intervention and associated transition coordinator

An automated messaging intervention and associated transition coordinator were generally viewed as an acceptable and appropriate approach to support AYAs during healthcare transition. However, participants identified several factors to consider in its design. Participants noted that individuals have varying preferences for communication methods. While some may appreciate phone calls, others may have an aversion to them. Offering various communication channels within the intervention and allowing participants to select their preferred methods, such as phone calls, emails, and text messages, may accommodate these diverse preferences and enhance the intervention’s appeal. Allowing participants to select how frequently they receive communication can tailor the intervention to match their individual needs and preferences, ultimately improving their engagement and satisfaction.

AYAs emphasised the importance of using a warm, inviting, and informal tone in messages. They suggested that communication should feel personable and relatable to avoid coming across as inauthentic or robotic. Including informal elements such as graphics interchange formats (GIFs) and emojis was seen as a potential way to enhance relatability and engagement.

The transition nurse coordinator was identified by participants as an important component to enhance engagement with the intervention and to provide added support for those that need it. AYAs expressed concerns about perceiving automated messages, such as texts, as spam, leading them to ignore or dismiss unfamiliar messages. They suggested initially sharing information through a credible source, like a website or email, to establish the legitimacy and trustworthiness of the phone number sending the messages. Another suggestion for improving trustworthiness involved integrating a human connection into the messaging system, such as meeting the transition nurse coordinator. This way, AYAs could conceptually link the messages to the coordinator, enhancing trust in the communication process – putting “a face to the name”. Overall, participants welcomed the concept of AYAs having access to a transition coordinator for additional support. They emphasised the importance of either meeting the coordinator during the recruitment process or at least having a written or video introduction to establish familiarity with the human connection behind the messaging system.

Participants highlighted important considerations for the adaptability, accessibility, and cost effectiveness of a messaging system including consideration for individuals who may lose their phone, thus requiring an alternative method for maintaining contact. Limiting responses to yes/no options was perceived by some AYAs as a possible barrier to engagement. It was suggested that using open-ended responses, where appropriate, could encourage more detailed and meaningful feedback.

### Education modules

The inclusion of education modules within Transition Compass was largely perceived as beneficial, and participants provided input on various design considerations. AYAs emphasised the significance of tone in maintaining engagement and effectively conveying information in education modules. While humour was appreciated for its ability to captivate attention, AYAs also valued straightforward, informative approaches that prioritise clarity and purpose. The importance of having HCPs advocating for and encouraging AYAs’ engagement with the education modules was also highlighted. Participants suggested the inclusion of accessibility features like sign language interpretation and high-contrast visuals, as well as providing written text along with visual aids such as infographics. To enhance engagement and resonance, AYAs highlighted the importance of going beyond surface-level information by employing a storytelling approach rather than a mere “information dump”. They suggested finding a balance between generic and specific content to ensure broad appeal and relevance to most users.

AYAs raised varied perspectives on using social media such as TikTok or Instagram as a platform for information and education. There was recognition of its potential for relatability but also concerns about its susceptibility to misinformation. Concerns were raised about potential embarrassment if health-related content unexpectedly appeared within algorithms among friends. One advantage that AYAs saw in social media was the ability to easily consume short, concise content. AYAs noted that this bite-sized format would make it easier for them to quickly absorb information and stay engaged with the content.

The importance of drawing inspiration from existing models while ensuring that the Transition Compass intervention offers a unique point of difference was emphasised by participants. However, they advised against reinventing the wheel, advocating for the use of relevant and pre-existing resources where possible. This may include adapting existing ideas to suit the specific needs and objectives of Transition Compass.

AYAs advocated for an approach to the educational modules that was both authentic and relatable. Opinions varied regarding whether the information should be delivered by an AYA or a HCP, with some proposing that having both AYAs and HCPs represented may enhance the relatability and credibility of the content. Additionally, emphasis was placed on youth-led design, highlighting the importance of materials created with or by young people. Participants also advocated for accurate branding of the education resources to reassure authenticity and trustworthiness, avoiding the appearance of spam.

A range of relevant education topics were suggested by participants. These included but were not limited to independence and responsibility, expectation management, managing uncertainty, attachment/grief, advocating/knowing your rights, burnout, sexual health, driving and risk-taking behaviour, mental health, finance, discrimination, alcohol and drugs, lifestyle (sleep, exercise, nutrition), and Medicare and health insurance.

## Discussion

This study explicates the complexity and variability inherent in healthcare transition for AYAs with CMCs. Key factors influencing this transition were linked to CFIR domains, specifically the characteristics of individuals, the inner setting, and the outer setting. These factors included the life changes AYAs experience during this period, the need to develop autonomy in managing their own healthcare, and the varying levels of knowledge, understanding, and expectations among HCPs and AYAs regarding the transition process, and AYAs’ needs and abilities. Additional determinants included communication barriers, disconnections within and between paediatric and adult HCPs, care disparities and gaps during transition, and the role of specialised transition services. A significant overarching issue is the absence of standardised policies, practice guidelines and procedures in Australia. The proposed Transition Compass intervention, which includes automated messaging support, connection to a transition nurse coordinator, and education modules, was generally well-received and considered as an appropriate and acceptable approach to support AYAs through the transition process, particularly due to its adaptability to varying contexts.

Previous studies have consistently highlighted the challenges associated with healthcare transition such as variability in transition timing and the impact of major life changes for AYAs on this process^14,34^. This research corroborates these findings, demonstrating that inconsistent transition practices contribute to gaps in care and unpreparedness among AYAs. Furthermore, the findings align with literature that alludes to the ‘falling off a cliff’ perception, where AYAs often experience a significant reduction in support and continuity of care upon leaving paediatric services^34,35^. Other research has explored the need to foster a sense of confidence among adult service providers to manage the complex care of the young adult and develop an autonomous young adult, whilst actively involving parents/carers^36^.

Prior research has identified several enablers that contribute to successful transition programs, including assessing transition readiness, developing AYAs’ self-management and self-advocacy skills, and incorporating a transition-focused role within the care team, typically filled by a nurse coordinator^37,38^. The involvement of a transition nurse coordinator is particularly valuable, as it provides dedicated support for AYAs and their families, alleviating the burden on other time-constrained healthcare providers^39^. Our findings align with the literature, reinforcing the favoured view of a transition nurse coordinator in assisting AYAs during the transition process. Recognising the challenges of making systemic changes across both paediatric and adult healthcare settings, the Transition Compass intervention focuses on building self-management skills in AYAs, which is an identified enabler in transition programs, and acts on the inner and outer setting CFIR domains.

Automated messaging services, particularly text messaging, has been shown to support the transition process by offering appointment reminders, providing educational content, and facilitating self-management and independence^40,41^. This approach is recognised for its cost-effectiveness through the use of affordable technology, potentially reducing personnel requirements and saving time and resources. Expanding the application of text messaging to facilitate healthcare transitions and exploring its effectiveness and suitability in this context continues to be an emerging area of research^31^. Transition Compass offers an opportunity to incorporate co-designed considerations into an automated messaging system, ensuring it meets the diverse needs and preferences of AYAs.

The findings generated in this study will be used to inform both the refinement of the Transition Compass intervention prior to commencing the RCT as well as informing future studies designing interventions to support transition. It has enabled us to consider how the intervention influences key determinants of the transition process to achieve our desired outcomes. The findings will inform the development of an implementation research logic model to guide the finalised design, implementation, and implementation evaluation of the Transition Compass RCT^42^.

For pragmatic reasons, a combination of individual interviews, dyadic interviews, and focus groups were used for data collection. Although the triangulation of mixed qualitative methods is acknowledged for its ability to provide a more in-depth understanding of a phenomenon^43^, it is also recognised that using different interview formats can introduce limitations, as each method may elicit data with varying areas of focus^44^. The application of mixed qualitative methods enabled us to gather insights from a range of stakeholders, which may have been restricted by a more rigid interview approach. Different formats were considered as different components of data which were triangulated and synthesised as part of the analytic process. The interview guides were not designed with a specific focus on capturing all the constructs within the CFIR. As a result, the data collected may not fully represent the prominence of each construct in relation to the healthcare transition process and the proposed intervention components. Given Australia is a unique healthcare system in some respects, our findings may not be inherently transferable to contexts outside of Australia. However, we also note that the transition from paediatric to adult healthcare is not a uniquely Australian concern. Insights from our study may help to provide guidance for other countries developing and implementing scalable interventions to support transition. Our analysis did not explore the differences in the characteristics of AYAs who preferred to manage their conditions more independently vs. those who valued parents’ continued involvement. This is an important consideration for future research.

Despite these limitations, the use of the CFIR during intervention design is a notable strength. Although the CFIR has more widely been used during or post intervention implementation^45^ this research contributes its growing application during the pre-implementation phase. By systematically exploring elements such as the intervention’s characteristics, the outer setting, the inner setting, and the individuals impacted, the CFIR helps ensure that all relevant dimensions are considered. Applying this process during the pre-implementation phase facilitates the development of a more tailored and effective intervention by highlighting potential barriers and enablers early in the design process. As a result, co-designed feedback can be applied to ensure Transition Compass is better aligned with the needs of the target population and the practical realities of the healthcare environment, ultimately increasing the likelihood of successful implementation and sustained impact.

This research reinforces the need for transition interventions that can be implemented within the complexities of the current health service landscape and that are focused on strengthening self-management skills, thus empowering AYAs to successfully navigate and engage with the adult healthcare system. Applying the CFIR during development of the Transition Compass intervention has provided valuable insights into key determinants influencing the transition process, enabling identification of obstacles and opportunities to consider during the design phase and demonstrating its value in the pre-implementation stage of healthcare interventions.

## Methods

### Study design

This qualitative study was conducted as part of a Randomised Control Trial (RCT) multi-phase project which aims to determine whether the Transition Compass intervention can improve post-transition engagement with specialist services, reduce unplanned and crisis-driven presentations, and enhance patient reported outcomes and experiences. Transition Compass builds upon a pilot that showed that a structured transition coordination programme run by a transition coordinator, offering tailored transition support including appointment management, was feasible and acceptable^30^. Guided by co-design methodology^46^, the Consolidated Framework for Implementation Research (CFIR)^47^, and the Implementation Research Logic Model (IRLM) framework^42^, this design phase of the project comprised engagement with a range of CMC healthcare professionals (HCPs) and people with lived experience to identify potential barriers and facilitators to Transition Compass implementation, refine the implementation strategies, and adapt the intervention prior to trial implementation. The reporting structure is based on the Standards for Reporting Qualitative Research (SRQR; Supplementary Table 3)^48^.

The CFIR provides a structure for understanding barriers and facilitators to successful implementation within specific contexts, ensuring alignment with the relevant context, and guiding dissemination to other settings^49^. Using the CFIR to prospectively explore potential implementation challenges prior to widespread program implementation allows for the identification of key areas that may benefit from refinement, ultimately increasing the chances of successful implementation of the program^45^. Alongside the CFIR, the IRLM framework was used to assist the planning of Transition Compass design and implementation^42^.

### Setting

The Transition Compass RCT is a national, multi-site project which will be conducted in three Australian states; New South Wales (NSW), South Australia (SA), and Victoria (VIC). The four participating sites include the Sydney Children’s Hospital, Randwick (SCH) and The Children’s Hospital at Westmead (CHW) in NSW, The Women’s and Children’s Hospital, Adelaide (WCH) in SA, and the Royal Children’s Hospital, Melbourne (RCH) in VIC. This intervention aims to enhance the transition experience and outcomes for AYAs with CMCs. The proposed components for co-design consultation include automated messaging support, connection to a transition nurse coordinator, and psychoeducation modules.

The Australian healthcare system is a mix of public and private services designed to provide universal healthcare. Medicare, the public health insurance scheme, provides universal access to healthcare, covering hospital care, GP visits, and subsidised medications. Private health insurance for those who are able to pay the necessary premiums can offer additional benefits like faster access to care and private hospital services. State and territory governments manage public hospitals, while the federal government oversees Medicare. Key services include primary healthcare through GPs, mental health programs, and aged care. In terms of timing of transition care, transfer to adult care typically occurs once a young person turns 18 or around the time of completion of secondary level schooling. However the age at onset of preparation for this transfer is highly variable among paediatric healthcare providers.

### Participants and recruitment

This research project has been approved by the Sydney Children’s Hospital Network Ethics Committee (2023/ETH01180). Participants signed an online consent form via REDCap outlining their consent to audio recording and transcription. Participants were informed of the nature of the study and any benefits or risks associated with participating in the study. We purposefully sampled AYAs with CMCs aged 16-21 and parents of AYAs with CMCs from community and hospital-based groups based in four states across Australia, encompassing a wide range of chronic conditions. We excluded AYAs experiencing severe mental health issues and have concerns about their capacity to participate as well as those who did not have the cognitive capacity to participate. Paediatric and adult HCPs working with AYAs with CMCs were recruited through the research team’s clinical networks from various specialties such as neurology, rheumatology, endocrinology, nephrology, rehabilitation, respiratory, renal, cardiology, neuromuscular, dermatology, and ophthalmology clinics from 13 hospitals across five states/territories. Adult HCPs were also identified via snowball recruitment whereby participating paediatric HCPs were asked to nominate adult HCP colleagues who they refer their transitioning patients to.

### Data collection

Between November 2023 and May 2024, qualitative data were gathered through virtual individual interviews (*n* = 15), dyadic interviews with parents and AYAs (*n* = 4 involving 8 participants) and focus groups (*n* = 15 involving 64 participants) (Table 7) until saturation of the main themes was achieved as determined by consensus among the study team based on preliminary analysis. Varying interview methods encouraged interactive dialogue and accommodated logistical constraints such as the availability of HCPs at specific times. Focus groups ranged between 3 to 10 participants and included either HCPs or AYAs and parents. Interviews and focus groups lasted between 21 min and 2 h 5 min. Prior to participating in the interviews, participants were provided information regarding the purpose of the study and details about what participation entailed and provided consent to participate.

**Table 7:** Number of participants per participant group and type of data collection method

	Individual interviews n	Dyadic interviews n	Total interview participants n	Focus groups n	Total focus group participants n	Total participants n
AYAs	1	1	3	4	14	**17**
Parent	-	-	-	2	8	**8**
Paediatric HCPS	5	2	9	6	28	**37**
Adult HCPs	8	1	10	2	6	**16**
Mixed HCPs	1	-	1	1	8	**9**
**Total number of participants**	**15**	**8**	**23**		**64**	**87**

*AYAs* Adolescents and young adults, *HCPs* Healthcare professionals, Mixed HCPs comprised of either HCPs who worked across both paediatric and adult settings, or a mixed group of paediatric and adult HCPs present in the same focus group.

Using a semi-structured interview guide with open-ended questions (Additional file 2), we explored participants’ experiences with, and attitudes toward, healthcare transition for AYAs and their perspectives regarding the proposed Transition Compass intervention. Interviews and focus groups were carried out by Transition Compass researchers, each of whom had experience conducting qualitative research. All interviews and focus groups were audio-recorded, transcribed verbatim by a professional transcriptionist, and reviewed for accuracy. To protect participant anonymity, transcripts were anonymised. Parents and AYAs received remuneration for their participation.

### Data analysis

We explored barriers and facilitators affecting the transition process using an iterative hybrid deductive-inductive thematic analysis approach as outlined by Proudfoot^50^. We used NVivo software to facilitate the coding and organisation of identified themes. The deductive analysis applied the CFIR as a coding framework^51^, while the inductive approach was guided by Braun and Clarke’s six-step thematic analysis framework to identify emerging themes related to barriers and facilitators in the transition process and to the proposed intervention components^52^. The first author conducted the deductive-inductive analysis in continuous collaboration with author MH and through regular meetings with the research team, ensuring quality assurance and comprehensive representation of the data in the findings. While we did not engage in wholesale member checking of data, we clarified specific elements of our findings to help shape the Transition Compass intervention, namely scripts of the education models and language used for the automated messaging component. The coding tree is available in Additional file 3. Findings were then mapped to the core elements of the IRLM^42^.

## Supplementary information

Supplementary information

## Data Availability

Datasets generated and/ analyzed during the current study are not publicly available due to were as they were collected after participants signed a consent form assuring them of confidentiality, but are available from the corresponding author on reasonable request.
