# Supplementary material for: Co-design of an intervention to enhance healthcare transition for adolescents and young adults with chronic medical conditions
Source: Npj Health Syst. 2025 Oct 1;2:36. doi: 10.1038/s44401-025-00041-4 (PMC13354253; doi:10.1038/s44401-025-00041-4)
Supplement: Supplementary file 1 — Supplementary information [file 44401_2025_41_MOESM1_ESM.pdf]

SUPPLEMENTARY FIGURE 1: ANALYSIS CODING TREES

Determinants of Transition

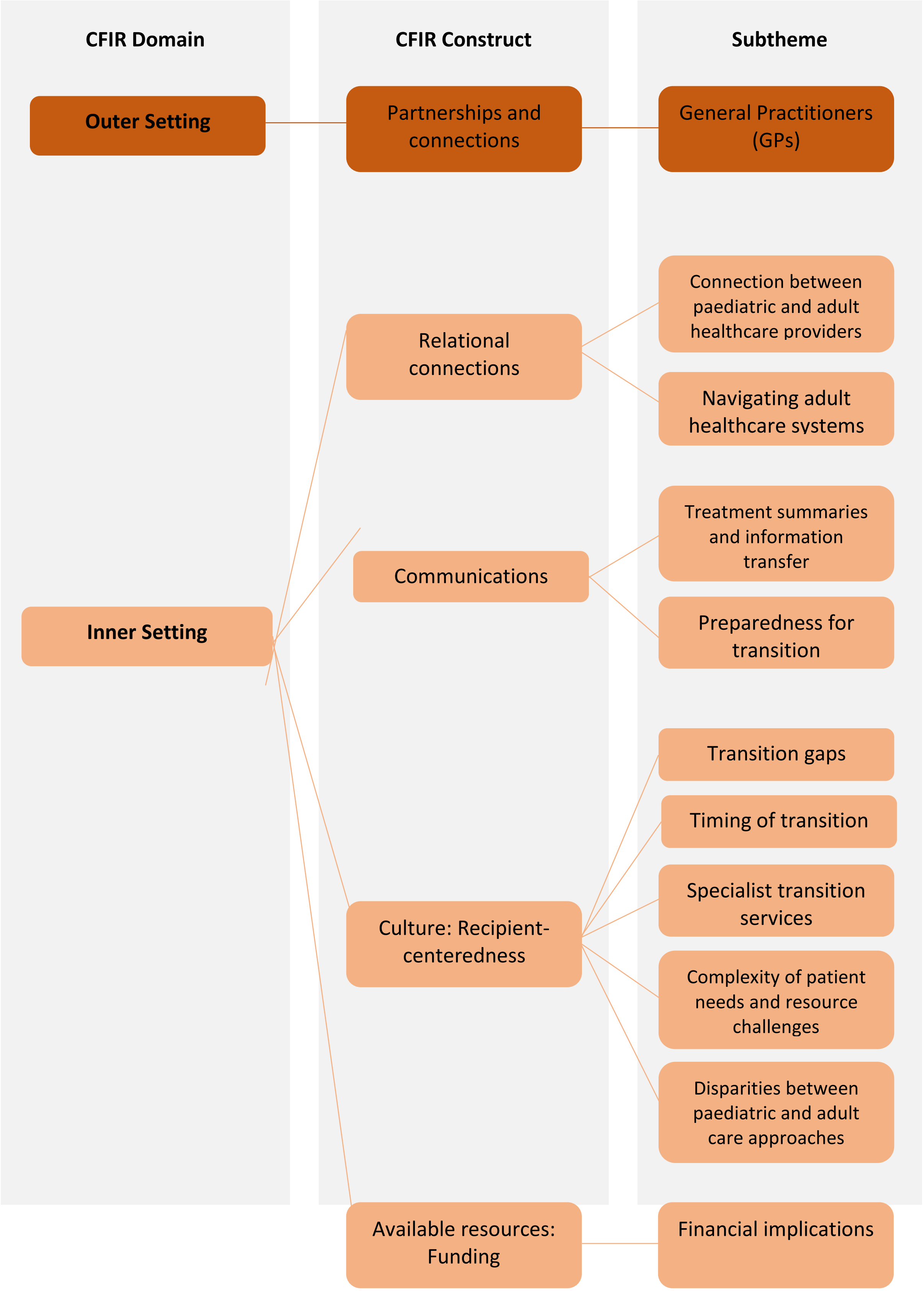

Determinants of Transition Continued…

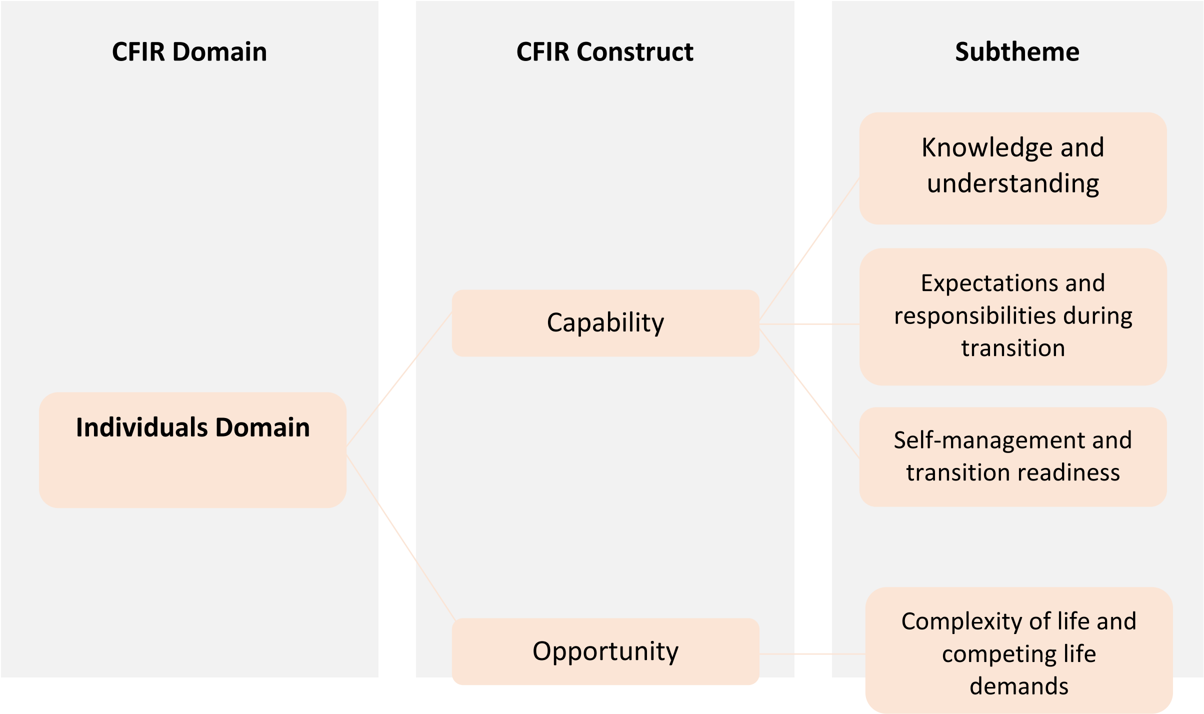

Proposed Intervention: Automated messaging intervention and associated transition coordinator

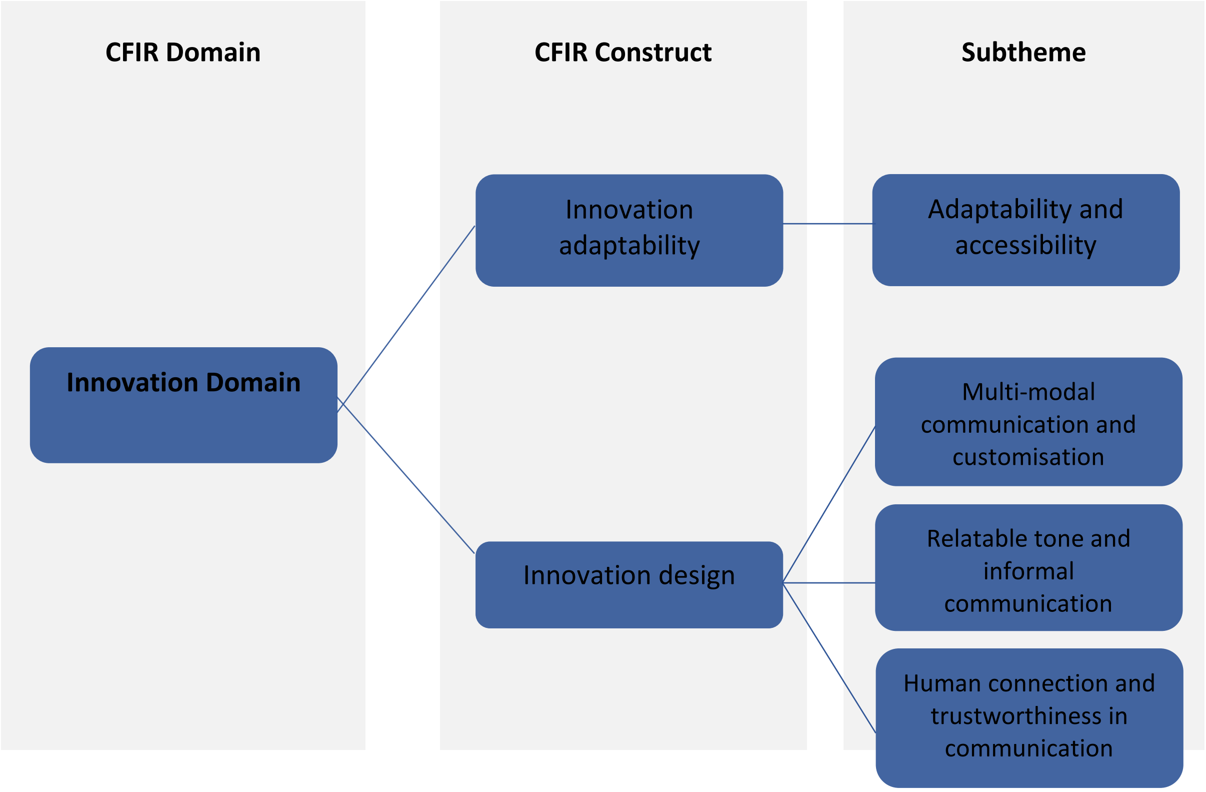

Proposed Intervention: Education modules

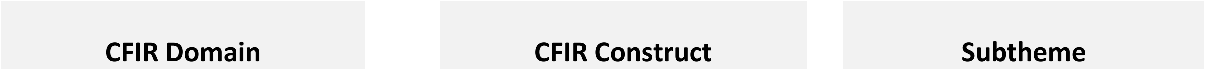

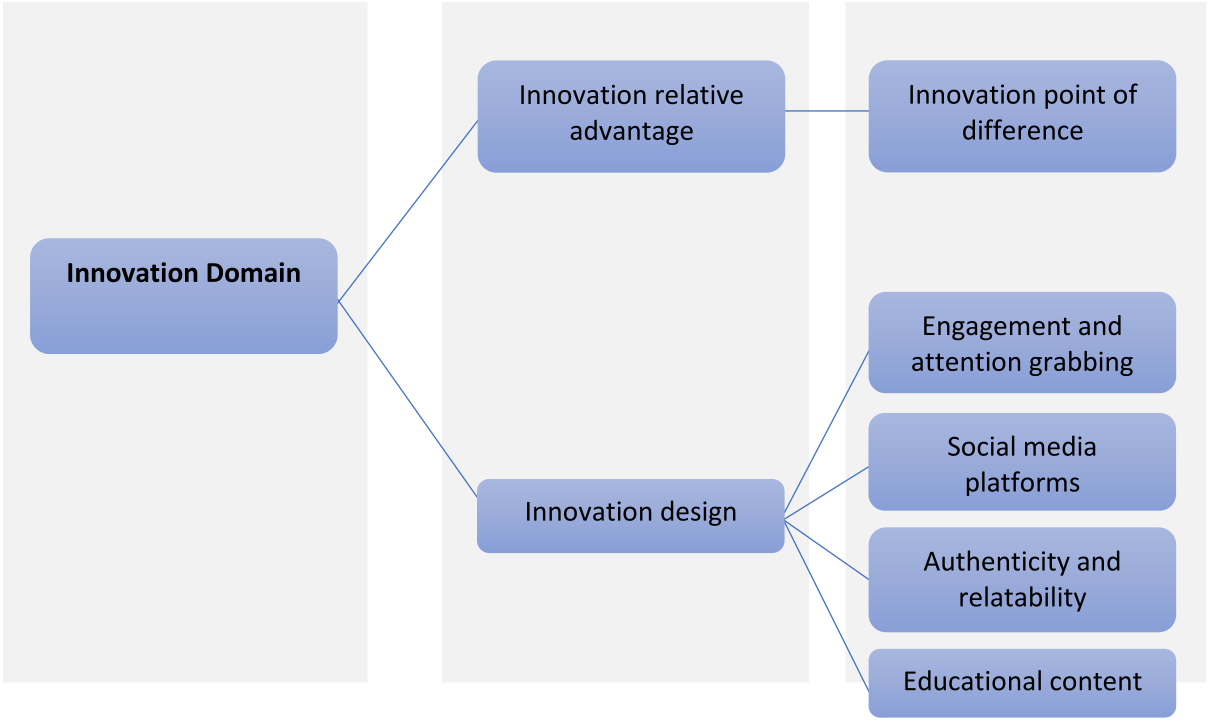

Supplementary Figure 1: Data analysis coding trees.

SUPPLEMENTARY TABLE 1: SAMPLE QUOTES BASED ON CONSOLIDATED FRAMEWORK FOR IMPLEMENTATION RESEARCH (CFIR) DOMAINS AND CONSTRUCTS, AND INDUCTIVE THEMES.

DETERMINANTS OF HEALTHCARE TRANSITION

**Outer Setting CFIR Construct**	**Theme**	**Supporting Quotes**
Partnerships and connections	General practitioners (GPs)	*"So, I just don't think it's as clear as to who's going to take care of this kid from a - there's no paediatrician. Paediatrician in some respects gets replaced by a GP, which is not sufficient, because these kids, you need to see them for an hour, like most of the time." Paediatric HCP 08*<br><br>*"So, we kind of were explained to that the GP would kind of become the person that you go to for everything in the person that kind of holds your healthcare team together. But I didn't even really have a regular GP at that point, because it's so hard to even get into see GPs… the paediatrician I had at the hospital had that knowledge of the kind of different disabilities and the different things that came with having a disability, whereas my GP, obviously is more general. So, doesn't have the same level of knowledge." AYA 14*<br><br>*"I think the trouble is too, with GPs now like, they're in medical centres, you don't often see the same GP all the time, or there's lots of changes." Paediatric HCP 11*
**Inner Setting CFIR Construct**	**Theme**	**Key Quotes**
Relational connections	Connection between paediatric and adult healthcare providers	*"I mean, it [joint clinics] sounds like such a wonderful concept. But it just doesn't work and hasn't worked since, you know, I did the clinic 20 years ago as a Fellow and it wasn't working then and it's worse now. And, you know, I think trying to work with our adult colleagues to actually set these clinics up, I mean, we send staff down there. And they don't use them in the way that would work." Paediatric HCP 21*<br><br>*"And just before Covid, we used to do actually joint clinics at the Children's [hospital] which I found excellent. So typically, [paediatric neurologist] would book a whole morning with patients lined up for transition. And we would see them jointly with him as their paediatric neurologist. And for the family, this would be an opportunity to meet their prospective adult neurologist ask any questions or clear out any concerns they may have. And then we would then see the same patients in the adult clinic." Adult HCP 07*<br><br>*"One thing I've always believed in is the concept of overlapping transition… [it's like] you're running a relay, you hand over the baton, then both runners run together holding a baton and then the person let's go. So, one thing I've asked the [paediatric hospital] registrars to do… is that the [paediatric hospital] team are not discharging from diabetes care until they know [the patient] has had their first [adult hospital] appointment. And that has been, I think, a positive step because we've had some terrible instances of people being left high and dry, they're discharged from*

		[paediatric hospital], they can't engage the [paediatric hospital] system, and they're still waiting for [adult hospital] to kick in." Adult HCP 13<br><br>*"So, I have, I've got a few things, but like the main one being arthritis, so I was lucky enough to find a rheumatologist who sees both children and adults. So, with her, I've been able to, there hasn't been any transition with her at all because I've just continued to see her." AYA 11*<br><br>*"I think that paediatricians don't know who is the best person to see their patient when they become 18. And it's really hard to find someone to ask. Adult hospitals are, you know, big busy places as children's hospitals are as well. But there's, I think there's a bigger range like paediatric compared to adult endocrinologist is a very different number of people. And I think it's hard to know who the best person to refer someone to. That also carries on to patients who get lost to follow up or get lost to that transition process." Adult HCP 05*
	Navigating adult healthcare systems.	*"I suppose barriers are things like where there's lots of teams involved, and care like in the paediatric setting, you know, anyone, we can see anyone across the state at [paediatric hospital], but in adults, it's very much based within your local health district [LHD]. So typically, care will be based there. But for some services and clinics, they might not run particular services. So that might need to be at a different hospital in a different LHD. So having care split up can be a real barrier to then successfully linking and engaging with adult healthcare because it makes it more confusing, there's more travel, it's more difficult to get familiar with the new environments." Paediatric HCP 09*<br><br>*"I have specialists across different hospitals, they never communicate together, or if they request medical documents, it never gets it through." AYA 13*<br><br>*"I think broadly speaking, there needs to be a generalist in the adult space who coordinates care, and then there needs to be a level of engagement amongst all those sub-specialties to provide ongoing sort of expertise or care. But there needs to be a pathway for someone to move from the paediatric to adult space with a level of coordination and an ongoing, I hate to use the word case management or case coordination, but for someone with a complex, for example, some of the complex disability, you do actually need a generalist to be providing an overall sort of holistic picture of care, because what happens is, it becomes very sub specialised. And things can go wrong, when people aren't sort of stepping back and looking at the whole picture. If you just put a cardiology lens over a person, it's, you could miss some other stuff." Adult HCP 06*
Communications	Preparedness for transition	*"I think for us, the fact that we didn't even know we were transitioning out at 18 was a bit of a surprise so, I actually would have preferred to have known, you know, whether it was a year or two prior to just to say, Hey, by the time she's 18, and she's left school, she also needs to leave paediatric, and go into the adult system. So, you know, and for someone to then start contacting us about what that would look like. And, you know, because it seems like there is a lack of communication." Parent 01*

		"You might actually be meeting the child for the first time on their last paediatric appointment, either because you're, you know, covering clinics for a colleague, or actually, they've been referred at a late age and then you're seeing them as they're essentially kind of preparing for transition." Paediatric HCP 22<br><br>*"And they [paediatric HCPs] are preparing patients as they near the age, you know, whether it be 16 or so, that transition is coming. And I think that's a really important thing, just to start discussing transition a lot, you know, a long way before the age of 18." Adult HCP 05*
	Treatment summaries and information transfer	*"So, some [treatment summaries], better than others. Some clinicians are fantastic. And they provide really good summaries. Could be, that letter could be a file, what they do, and they're actually some are very good at this, they give the whole file to the family not to the clinician, because they're worried that things will get lost, so at least, you know, the family always had, we always have that file. And some others more succinct. So, it does depend on the individual." Adult HCP 07*<br><br>*"I think some other challenges is that there's no kind of standardised process by which transition occurs in Melbourne tertiary centres or I think, nationwide. There's no transition tool that clinicians can use to standardise that process. So, it's really, you know, what that particular paediatrician thinks is important to hand over to that adult clinician, and, you know, having worked on the paediatric side, you don't have, you know, all this time to be writing pages and pages essays of information to hand over - you've got lots of you've got busy clinics, a lot of patients are transitioning." Adult HCP 05*<br><br>*"Yeah, most of the time the records are sufficient, where there's I guess, gaps and stuff, then we naturally actually repeat those things." Adult HCP 10*<br><br>*"I don't think a simple letter kind of can encompass an 18-year period of a kid's life." Paediatric HCP 25*<br><br>*"A strength that's continuing to develop is, in our precinct, the common electronic medical record between the [paediatric hospital] and the [adult hospital]. That certainly gives us much greater confidence in identifying the history and the medical history of the patient. That's a great help as we transition." Adult HCP 16*
Culture: Recipient-centeredness	Timing of transition	*"I think yeah, giving them enough time, because, you know, just, you know, not leaving it too late to transition them either, you know, 18 or 17 and three quarters and then transition them, some patients may need to start that transition process a little earlier. And that has to be driven obviously by the paediatric team, really, to know those patients, won't be for everybody. But, yeah, just introducing slowly where needed could be helpful as well." Adult HCP 08*<br><br>*"And that was another thing that I decided a few years ago was when I tried to transition them when they were in Year 12. They all said I'm gonna do this or I'm gonna do that or they're saying I'm not sure if I'm gonna do this. And so*

		I just say I'll see you in the February after when you've got the [HSC] results and you've decided to uni and then we know where you're gonna live." Paediatric HCP 17
	Transition gaps	*"I just dropped off, the paediatrician dropped out of the children's system that was it kind of like they were like, Okay, you're an adult now… And I had a few emergency admissions as well, during that kind of transition period, one in particular when I was 17." AYA 14*<br><br>*"Sometimes, it's unrealistic the gap of when they [adult services] according to their waitlist, or when they're going to actually review and see them, we might do another interim one [appointment], just to tide them over." Paed HCP 07*<br><br>*"I'm not confident that the loops been closed, and we've seen all of the ones that have been handed over for transition. And there are some that for some reason haven't come." Adult HCP 16*<br><br>*"From paediatric service for the adults, I think, ideally, we [paediatric HCPs] would know that our job has been done, that there'd be communication back to us. So, my experiences, anecdotally, that sometimes happens, sometimes it doesn't. So, it's nice when it does happen." Paediatric HCP 22*<br><br>*"I used to see a neurologist. And it was near impossible to actually find someone because the age that I needed to actually go and find someone I was around 15 or 16, like when my health got really bad. And literally everywhere, any paediatrics specialists were saying all now you're a bit old to now join the child system. But then they referred me to adult systems who were saying, you're only 15 or you're only 16 we can't take you on. So they're a long time, where actually wasn't getting any help at all. Because they was saying, 'Oh, you're around the age where usually we'd start transitioning kids into the adult system.' Whereas I needed to see someone in the first place." AYA 11*<br><br>*"I'm also like very small like for my age. So, my disability means that my muscle mass is a lot less. So, I need a lot of I need basically all paediatric equipment. So, for me to be in the adult department was really impractical." AYA 14*<br><br>*"When you transfer you transfer at different ages, depending on each department, which can be difficult. So for example, if you went to like a chronic pain service, and then a dental service, and then a cardiology service, one might transition at different points to another. And that can be difficult for people to go to multiple different places and be accessing paediatric and adult at different stages for each different condition, which can be difficult when you're dealing with multiple conditions that can affect the other ones. So, a change in a treatment from one condition might affect the treatment for another condition." AYA 16*
	Specialist transition services	*"And then from a neuromuscular clinic, we definitely involve [transition service]. So they're often very complex kids who are weak and have respiratory doctors and spinal doctors and endocrinology for the bones. And so because there are so many specialists involved, they're the ones who our CNC [clinical nurse consultant] usually triggers this 'oh, you know, they're 16, should we start referring them to [transition service]' and then the [transition service] gets in contact with us, and we move it from there." Paediatric HCP 08*

		"[Transition service] does a great job. But every now and again [transition service] like you know, like the member of staff might leave and you know, there's, if something falls through the cracks." Paediatric HCP 24<br><br>*"And there are things like for example, like [transition service]. They're acting as a conduit to going somewhere. And for many people that need to be transitioned, they don't want to be spending that time with, for example, a social worker or an adolescent medicine person who's not going to look after their disease, it feels like it's a time waster. There are absolutely cohorts that can go there, the vulnerable patient that's got multiple problems and needs distribution to multiple places. But the functioning diabetic who needs to just get to adult diabetes services transition adds a layer that's not doing much for them." Paediatric HCP 17*
	Complexity of patient needs and resource challenges	*"A lot of mental health problems and a lot of developmental like autism, ADHD. I mean, and then some with more unusual, like chronic medical comorbidities as well. Yeah, there's a lot of a lot of mental health actually, a big one as well in the diabetes adolescent is eating disorders." Adult HCP 15*<br><br>*"Waitlist is a huge factor… So some of the spasticity clinics in other areas have a huge waiting list. So that's another factor is that people can't get in regularly. They don't like the fact that they don't have the same level of access as what they had in the paediatric services. Then you've got people in the regions who, in rural areas who would need to travel a very long way to have you know, if we're talking about Botox treatment." Adult HCP 03*<br><br>*"Let me talk about mental health. Impossible. So if you have, if you're an adult with an intellectual disability, or developmental disability, it's very, very difficult for you to access a psychiatrist. I would say at least eight to nine times out of 10, for any of my patients, they are declined by mental health services. And that's often so private, private psychiatrists will not see them… most of the mental health services will decline referrals for a patient with an intellectual disability, stating that it's not their skill set." Adult HCP 06*
	Disparities between paediatric and adult care approaches	*"I think the main thing that I felt was different from, you know, the paediatric adolescent care to adult care was the I guess the sense of like the quality of care and like the sense of personalisation. I remember like, when I was in paediatric care, I was in care, if I ever had any questions or anything, I would be able to easily be able to get in contact with a nurse manager or like my doctor or something, and get really quick answers for like, any questions that I had regarding like, symptoms, or conditions or progress or next step, and it would be super easy. And now that I'm in the adult system, it's like, oh, yeah, you just have to basically bundle up all your questions. And just, like blurt it all out in the next consult that you have with the doctor. Which was something that I never knew that, like, the level of quality of care would drop so much comparatively." AYA 09*<br><br>*"At the Children's Hospital, they get all the specialists kind of almost in the room together. Well not necessarily specialists, but the OTs and allied health would be in the room with the paediatrician. And then I don't know, everyone just seemed to know what was going on. Whereas in the adults, they kind of expect you to tell your story and then you see someone different every time as well, which makes it really, really difficult." AYA 14*

		"So, one of the biggest barriers we find is that in paediatrics, there's some publicly funded services that do not exist in adults. And a really big issue is psychiatry, especially for people with ADHD. So, there's no publicly funded adult clinic – in paeds a paediatrician or psychiatrists can provide stimulant medication, but in adults, it needs to be an adult psychiatrist. There's long waiting lists is extremely expensive. So that's a real barrier, especially for families of lower socio economic, there's really no cheaper option." Paediatric HCP 09<br><br>*"Something that will be like 'what happened to this kid?' and they'll be like, 'oh, they didn't engage so we [adult services] discharged them' and that is not an uncommon thing. Whereas in the paediatric space, we'd have that capacity to follow up and kind of get to the bottom of, like, reasoning behind that." Paediatric HCP 15*
Available resources: Funding	Financial implications	*"I don't get bulk billed anymore. Usually, unless I really emphasise my soul story, which is that I get paid. I work full time, but I get paid $13 an hour as an adult, so an $80 GP appointment is almost a whole day of my pay. My specialists' appointments are usually more than a week's worth of my pay." AYA 05*<br><br>*"Yeah, another barrier is medication. So a lot of medication that is at low or no cost at Children's Hospital often is not available through the hospital dispensary - families have to pay a lot more money for the same medications." Paediatric HCP 09*<br><br>*"As soon as you are both over 18, and not a full-time student, you are no longer covered by your parents' family ambulance cover. And you have to get your own ambulance cover. So, say for me, I turned 18, right before exams. As soon as my exams were done, I was no longer covered under my parent's ambulance cover I had to pay for that myself." AYA 05*<br><br>*"So often they're [AYAs] working multiple jobs and skipping on meals, food quality. And with diabetes, it's very day to day decision making stuff. So, I think there's a very direct impact. We have quite a few people worried about paying rent and they can't afford medications." Adult HCP 03*
**Individuals Domain CFIR Construct**	**Theme**	**Key Quotes**
Capability	Knowledge and understanding	*"And then they've got this very rare condition, which I kind of looked up there – had never heard of it. And they have some bone issues related to that with osteoporosis. But I don't think that bone issues are connected, but I'm not sure. You know, just. So, then I know that they've seen they said that they had seen a very particular paediatric genetic bone specialist at the Kids Hospital." Adult HCP 04*<br><br>*"One of the things that frustrates me a lot is when you ring up or like say, 'Hey, I have this condition, are you knowledgeable about it.' And they go 'Oh, yeah.' And then they say things that are like, you have absolutely no idea what the condition is. Because if you did, you wouldn't be saying this to me." AYA 01*

		"In the adult hospital when you go to emergency, actually, no one knew what my condition was. And it's not like it's rare. It's just yeah no one knew. So when I'm at my most sickest, instead of just being like, I need this and this, I have to go back and explain the whole thing again." AYA 13<br><br>*"So, I like the transition, as I am taken more seriously as an adult. The trade-off is that I'm expected to make my own decisions rather than deferring to my carers, whom I still rely on for support. It would be good if medical service providers could be more empathic in this regard when dealing with young adults, especially those who have just transitioned to adulthood." AYA 16*
	Expectations and responsibilities during transition	*"Well, yet, I mean, the main thing is just understanding that it's [adult care] a different beast, and people that they'll [AYAs] come across will be much less familiar with their conditions. And people in the adult system are more used to looking after ageing healthcare problems. And so, it's important that person advocates for themselves and doesn't necessarily just assume everybody knows what they have." Adult HCP 02*<br><br>*"Mum, and I were kind of like it was clear that it was expected for me [in adult services], myself and family to become like the people that talked to all the specialists and kind of connected all the dots, if that makes sense. Because I feel like, I think they intend for that for the GP to do but it's really not at all, it wasn't my experience anyway. And it really hard to like, understand where you're where to go for different things. And just I feel like I didn't have the confidence knowing that everyone was working towards the same goal." AYA 14*<br><br>*"I think they're [AYAs] quite fearful. The feedback that I get in the transition clinic is that they, they're quite anxious. They've been, particularly people who've lived with a chronic health condition. In the paediatric services, they're very well supported. It's very well coordinated. And there's a lot of discussion from people that they know about how fragmented and siloed it is, once they get to the adult space…So, I think the fear of things being different, the fear of things being not as well supported at a point where they're trying to develop their own autonomy and independence and manage being a young person, as well as a health condition. A lot of people are transitioning. So it's quite a busy time for them. They're transitioning to, from school to, to study or vocational programmes, and that there's a lot going on, and I think it then just becomes too hard." Adult HCP 06*
	Self-management and transition readiness	*"For me, I was always very into being independent about my medical health, I always wanted to talk to the doctors, I wanted them to talk to me, I didn't really want my parents to be involved too much. I wanted an acknowledgement that I was, it was going to affect me at the end of the day. So talk to me, not my mum, not my family, talk to me, because it's my body." AYA 12*<br><br>*"Especially for those of us with developmental disabilities, super essential to have doctors supporting our parents and carers being in appointments." AYA 19*

		"I've ended up getting, like using Notes on my phone… in the few weeks leading up to the appointment, I have a list of like, if anything's happened, and then like, things I need to ask and things I need to tell her about. Whereas beforehand, like, I'll admit, when mum went to appointments with me, I'd sit there in silence. And whenever the doctor asked me a question, I'd just look at mum and wait for her to answer for me. Like, she's always been the one to like, answer for me. And then all of a sudden, she's like, no, you're old enough. Go on your own. So like, I sort of have to talk for myself now. Which is terrifying. And I have no idea what to say. So, like, making a list beforehand helps." AYA 10<br><br>*"But look, it takes time to build that relationship with the family for how efficient or effective that the transition clinic or the transition model is, in general. These are patients and families who have spent potentially two decades with the same team, and now they are trusting a new person they've met once or a couple of times, you know, we taking care of the health and the condition of their loved one, of their child. So it's a very difficult process, it does take time to establish that relationship of trust." Adult HCP 07*
Opportunity	Complexity of life and competing life demands	*"So yeah, even just the frequency of appointments and also as an adult, you know, you often not all the time, but often you do have full time work or other commitments, when as a kid, you know, the school which is important, but it is a little kind of easier to get around. And so I think the frequency of appointments impacts life a lot more I find." AYA 06*<br><br>*"There are many really valid reasons why young adults fall through the cracks, you know, they might be starting university, they might be starting work for the first time, they haven't got parents that are prodding them to make it to their appointment, they can't drive because they're having seizures." Paed HCP 04*<br><br>*"And as carers of our children well, they're trying to get their independence and break away from their parents in a nice way. But they're also having the issues were having on top of all their young adult issues, like their mental health. And a lot of them are wanting to start a new career, education, on top of their disability, so and then accommodation, they're probably looking at moving out of home. So they're the sort of, they've got a lot of issues as well, that need to be looked at and I think, you know, the transition team should be looking at that as a holistic approach as well." Parent 03*
**Innovation Domain CFIR Construct**	**Theme**	**Key Quotes**
Innovation adaptability	Adaptability and accessibility	*"I'm thinking of kids that lose their phones, prepaid phones" Paed HCP 06*<br><br>*"Having two points of contact for them. So, like mobile numbers can go down and having a backup email or whether it's a or Instagram, and like they'd give it to you." Paediatric HCP 18*

Innovation design	Multi-modal communication and customisation	*"I think you'd have to consider multimodal, because for some, yep, they'll use their phone and they'll possibly be able to follow through on what you're offering. And for others, that's not going to be probably a useful pathway. Paed HCP 05*<br><br>*"It would be great to have an option. Like when you send out your initial email, you could say, hey, how often would you like us to contact you, and then give options weekly, monthly, fortnightly. And then they can decide." AYA 01*
	Relatable tone and informal communication	*"I think it's also important to kind of watch the tone as well, because I think as I do kind of have a bit more hesitant to click on a notification. If I sense the tone is a bit more serious, or it's a bit more cold, I say. Not really aggressive. But yeah, kind of like really upfront. I do kind of tend to avoid that just personally." AYA 03*<br><br>*"I think it depends on your target audience. Like if you sent me a text like that, I would probably not be a fan. But like some people, like that's how they talk. And, it depends if it's someone trying to act like a teenager, and they missed the mark. And it's like, probably won't really appeal to many people." AYA 01*
	Human connection and trustworthiness in communication	*"I don't really click on messages that are 'Hey, [AYA 10]' because I just think it's spam. So I think if you first disseminate it via like a website or email that like legitimises it, so then people can be aware, like, Okay, this is a legit number that I'm receiving the text from." AYA 10*<br><br>*"With texts, it's more likely, like I'm more likely to just dismiss it as spam." AYA 11*<br><br>*"I definitely think that's much better considering like you've given her your contact information. So, there's like a face that you can put to the name. I definitely think that would be much better as opposed to just like some random automated situation." AYA 10*
**Innovation Domain CFIR Construct**	**Theme**	**Key Quotes**
Innovation relative advantage	Innovation point of difference	*"I just wanted to let you know we've got some [educational resources] already. So, we have an education video for teenagers, it has teen actors in it, it was researched based on all of the safety messages and education they require before they leave... So, you wouldn't have to reinvent the wheel on those resources they already are available." Paediatric HCP 05*
Innovation design	Engagement and attention grabbing	*"I feel like you can do this in a variety of different ways. But I do really like the tone in that last video, like it's really clear. Kind of soft, but powerful, as well. I liked the first one because I did like the bit of comedy when all the files were dropped. So, I think a bit of humour, a bit of comedy is always great. The, like documentary style one feels like when you have someone who talks like the same, it loses you. Like, I feel like there needs to be fluctuation in the way people talk to keep people engaged." AYA 01*<br><br>*"And then on top of that, as well, I think it's very important to have like a ground approach with the staff. So, they're actively involving and pushing, you know, young clients and young patients, towards the modules. And that could be*

		you know, as something so simple as you know, a doctor or nurse has been seeing the same patient for a while and noticing that, you know, they might have some concerns about treatment, and then be like, 'Hey, here's a module blah, blah, blah', having a quick discussion about it with them. Yeah, cause I think if people find out on the ground, like face to face, that's also very important." AYA 09<br><br>*"I think it's always better to make it a story instead of an info dump. Because info dumps never really worked really well when it comes to human videos." AYA 09*<br><br>*"I liked hearing about the personal experiences and stories." AYA 10*<br><br>*"But once again, I think having like, you know, 20 young people in a video, the more people you showed, the more like dilutes the actual story or the meaning that you could convey, you know, maybe instead of like, focusing on 20 people focus on one person's story. And that actually, you know, means something in the end." AYA 09*<br><br>*"And you have it in a way that like it's presented, legibly, and really easily. And I think with videos, it's probably good to note that it would be great to have like an AUSLAN Interpreter. And when you're thinking of making videos, if you're doing like an animation style one with text and stuff, try and think of like the high contrast, and some of the different accessibility features." AYA 01*<br><br>*"I think that the background and the text might be a little bit hard to read. I personally prefer videos. Some people prefer written text. So I think that's something you have to be really mindful when you're creating different things, I think if you do go with written text, it'd be really great to have like, diagrams and present have written text for people who like to process information by reading, but have diagrams or like infographics and things that people could just glance at and sort of get the gist, because not everyone would read that." AYA 01*
	Social media platforms	*"I think TikTok is like a great place for relatability. But I also think that TikTok is a huge rabbit hole of misinformation. I actually worry about like possible embarrassment, like, if someone's with their friends on TikTok, and this suddenly comes up, they might just feel it a bit like, I didn't want my friends to see that." AYA 12*<br><br>*"I'm definitely more likely to just scroll through, like, many little videos, whether it's on like TikTok or Instagram or something, rather than read like an essay." AYA 11*
	Authenticity and relatability	*"But just, I feel like my main point is as long as it's like, whether it's like made by young people or definitely made with young people, so like, they, it's a youth led project. So I think that's really important." AYA 01*<br><br>*"But if you have, like two health professionals, like, just start discussing about it really in depth, that's really interesting to listen to, as well, because they really know, their topics and what they're on about. Yeah, cause I think, like, I find myself like listening to stuff that, you know, I would never listen to or don't know about." AYA 09*

		"I think maybe just like incorporating more people who actually have like those diagnosis, or maybe actually trying to get an actual young person who is transitioning out of care, and their experience into the videos, because it feels a bit weird having someone who's like, you know, 25 or something speak about transitioning to adult care when, you know, they probably haven't done it in ages." AYA 09<br><br>*"Yeah, if it like, how, if you advertise that, I don't know if it is or not, if it was, like, partnered with UNSW. And I feel like some people can, like just claim that title as well. Like, as long as like, you have, like, feel like a website's a good idea so they can check that out this text message. And I feel like Yeah, as long as like, you show in some way throughout that it's like a legit thing, because some people could just take UNSW, they could take like your idea and just do a like complete, like, fake one, as well. So there's a lot of like, creepy scans that are very, they look very realistic as well."* AYA 01
	Educational content	*"I always think of epilepsy is a bit like HIV - it was the disorder that hit every aspect of life. So for these teenagers, it's an issue of lifestyle. Sleep, alcohol, driving, pregnancy, because the drugs are an issue, contraception, SUDEP [sudden unexpected death in epilepsy], there are many, many issues that require education."* Paediatric HCP 03<br><br>*"I think some of those lifestyle things, you know, smoking recreational drugs, alcohol. Yeah, I think that's probably, you know, sexual health, mental health, that's probably more pertinent for that age group we see."* Adult HCP 06<br><br>*"Money management. Yeah. That's really important, isn't it when you become an adult and have to manage all these additional health costs?"* AYA 04<br><br>*"I think another thing is because the transition is so big, mental health literacy, and that recognition of the impact on that age group is quite a lot. So I think some resources around who to contact and what anxiety and depression might look like would also be helpful as well, because I think that change in circumstances can be quite significant."* Paediatric HCP 23

SUPPLEMENTARY TABLE 2: HEALTHCARE TRANSITION PROCESS PATHWAYS FROM PAEDIATRIC TO ADULT HEALTHCARE SERVICES

Paediatric department	Timing of transition	Awareness of upcoming transition	Estimated AYA transitions per year	Transfer summary completed by paediatric specialist	Referral to specialist transition service	Paediatric-adult connections	Transition gap between paediatric and adult services	Other
**Dermatology (Site A)**	Not specified.	Not specified.	~ 5	Not routinely required due to self-referral.	Nil referrals to specialist services	Works across paediatric and adult services so primarily refers to self.	AYA may present to ED.<br><br>Paediatric HCP may allow additional appointment if delay in adult services.	
**Endocrinology (Site B)**	Begins conversations and/or transition process around age 16.	Variable / no set process. When an AYA attends an appointment during their latter teen years, the clinician may decide on the spot to transfer them.	~ 15	Routinely completed.	May refer to transition service for complex cases.	Well-established young adult diabetes clinic.<br><br>More difficult to find other endocrine specialists.	May allow one more visit on an ad hoc basis while waiting adult services.	Attempts made to provide an educational session prior to transitioning out but are only able to do this if they are aware the AYA is coming up for transition.
**Endocrinology (Site C)**	15-16 years for Young Adults Diabetes Clinic (YADs).	Usually aware of AYA's coming up to transition age: linked to school completion or turning 18.	~ 100 with diabetes<br><br>~ 50 with other endocrine conditions	Routinely completed.	May refer to transition service – usually for complex patients.	Transition to YADs for diabetes patients.<br><br>Limited knowledge of	Intermittently may allocate precautionary appointments for AYAs in case the waitlist for adult services	

	17-20 years for other endocrine patients.					other adult specialists in the adult sector.	exceeds the appropriate timeframe for the AYA to be seen.	
**Endocrinology (Site D)**	Begin the transition process around school-age Year 12 (17-18 years).	No formal process but can sort patients by age which would allow identification of those aging towards transition.	~ 10-15+ with diabetes<br><br>>15 with other endocrine conditions	Routinely completed with inclusion of social vignette.	May refer to transition service for complex cases.	Joint clinics trialled but faced barriers.<br><br>May ring adult specialist if patient is particularly vulnerable.	Paediatric HCP continues care until AYA seen by adult services.	Mixed proportion of AYAs transferring to GP or specialist adult care.
**General paediatrics (Site E)**	Begin looking at transition around 16 – 17 years.<br><br>Able to see AYAs up until age 18.	Not always clear that it is the AYA's last paediatric appointment.	Not specified.	In the process of transitioning to electronic records.<br><br>Currently, paediatric information can mostly be accessed by adult setting.	Not specified.	Good relationships between paediatric and adult services.<br><br>Some joint consultations provided between paediatric and adult endocrinology services.<br><br>If capacity allows, someone from paediatrics will attend the first adult appointment with the AYA.	Often receive phone calls from 19–20-year-olds seeking assistance for navigating systems, booking travel, and organising medications, which the paediatric team will support.	Has some fly in/fly out and visiting adult specialists.

**General paediatrics (Site C)**	Ad hoc process.<br><br>Efforts are made to start transition discussions and promote GP involvement as early as possible with long-term patients who have established rapport and relationships.<br><br>The initiation of the transition is more fragmented for patients who have infrequent interactions with paediatric specialists or experience inconsistencies in the specialists they see.	No formal process.	> dozens	Inconsistently completed due to time and resource constraints.	May liaise with transition services for support with knowing where / who to refer the AYA to.	Typically refers AYAs involved with the neuro development disability team in paediatrics to the adult rehab service at adult hospital who have a dedicated young adult complex disability service (YACDS).	Will sometimes continue to see AYAs beyond 18 if they're yet to be linked in with adult specialists.	
**Kids Rehab (Site B)**	Begins transition conversations around age 15.	Typically connected to an annual review, therefore, it is often identified at age 16 that the patient's next appointment will likely be their final one with paediatric care.	~ 100	Inconsistently completed by different doctors.	May refer to transition service for complex cases.	Carries out joint transition clinics between paediatric and adult specialists where possible.<br><br>Beneficial when able to refer AYA to specialists who work across	Not specified.	

						both paediatric and adult services.		
**Neurology (Site B)**	Begins transition conversations around age 16.	Within power chart, can identify who is due to transition across the following year.	~ 5-10	Routinely completed and includes relevant clinic letters.	May refer to transition service for less complex cases.	Ad hoc co-consultation with adult neurologist and GP.<br><br>If the AYA is unable to attend the most commonly used adult service, the paediatric specialist liaises with adult colleagues to identify a suitable adult specialist in desired location for AYA.	Family may reach out to paediatric specialist for support - paediatric specialist encourages families to follow up with adult services.	
**Neurology (Site D)**	Begins transition conversations around age 14.<br><br>Able to see AYA up until age 18. (however, some may transition earlier).	Not always clear when the AYA's last appointment might be, but likely between 16-18 years.	Not specified.	Routinely completed and includes relevant clinic letters.	May refer to transition service.	Rare, ad hoc co-consultation for complex cases.	Paediatric specialist may see AYA if there's a delay in being seen in adult services.	Limited number of epileptologists to refer to within state.<br><br>Paediatric clinicians provide an adolescent day inclusive of education.

**Neurology (Site B-alt)**	Begins transition conversations around age 16.<br><br>Aims to transition before or after final school exams.<br><br>Adult setting may take them between 16 – 18 years.	Each time an outpatient clinic is held, patient lists get printed which allows for identification of AYAs who are due for transition.	~ 5	Routinely completed and includes relevant clinic letters.	May refer to transition service for complex cases.	Difficult finding suitable adult specialists.<br><br>Attempts made to have handover conversation with adult specialist for complex patients.	Not specified.	If paediatrics receives a referral for a 16-year-old, the referral is often forwarded directly onto an adult specialist to avoid the transition process.
**Neuromuscular (Site B)**	Begins transition conversations around age 16.	Runs two transition clinics twice per year which is typically the AYA's final appointment with paediatrics.	~ 16-20	Routinely completed.	Refers ~ 95% to transition service.	Structured neuromuscular transition clinic run twice per year involving paediatric and adult specialists.<br><br>Transition clinic not offered for other conditions such as respiratory and cardiology.	Not specified.	
**Ophthalmology (Site B)**	AYA remains with the service until 16 – 19 years.	Not consistently known that it will be the AYA's final appointment.	~ 20-40	Formal discharge letter or letter of transition frequently completed.<br><br>Certain adult hospitals have a referral	May refer to site B transition service for complex cases.	Beneficial when able to refer AYA to specialists who work across both paediatric and adult services.	Paediatric team may continue to see the AYA on an ad hoc basis while waiting adult services.	AYA's often forced into the private (and more costly) sector due to limited options in public sector.

				template to be used.				
**Renal (Site C)**	Introduce the concept of transition around 15-years.<br><br>Most transition around 16-17 years but for some, up to 18 years.	Transition clinics are scheduled around July/August and then November/December, with the goal of having the final appointment ideally just after Year 12 exams.	~ 10-15 into tertiary centres.<br><br>Mixed proportion will end up in community or GP follow-up.	Open letter routinely provided.	Not specified.	Transition clinics with local adult services once or twice a year from around age 16-years.<br><br>Attempting to develop a pool of adult nephrologists to access beyond main city centre services.<br><br>Epic medical records allows for adult hospital to access paediatric medical records.	Continue to provide care until the AYA is seen in adult services.	Transition coordinator available.<br><br>The adult hospital has a designated intake clinic in February for the transitioned AYAs.
**Respiratory (Site C)**	For patients with cystic fibrosis, the transition process is initiated at age 15, with the transfer occurring between 18-19 years.	Have awareness of final appointments.	~ 12 for cystic fibrosis patients.<br><br>~ 20-25 VRSS patients.	A transfer letter is often completed but it's challenging to adequately represent 18 years of medical care, therefore attempts are made to share imaging and	May refer to the transition service at site C for complex cases.	Usually know the adult specialist they are referring the AYA to.<br><br>A transfer meeting might be held with a clinician from adult hospitals though they	Paediatric team may continue to see AYA if there's a delay in adult services.	

	Patients suitable for the [state based respiratory service] are referred around age 16, with a transition clinic appointment scheduled during their 17th year.			the accompanying reports.<br><br>Smoother transition to adult hospital due to shared access to medical records via Epic.<br><br>Adult services don't have access to paediatric medical records for cystic fibrosis patients.		may not be the long-term clinician taking over the AYA's care.<br><br>Despite a handover and a transition plan, the AYA may still be left without a confirmed adult specialist appointment. The paediatric clinician will continue to see the patient to avoid any gaps in care, but the initial handover information becomes outdated.		
**Rheumatology (Site C)**	Provision of a pre-transfer clinic with RMH nurse at the end of Year 12.<br><br>As soon as they turn 16, paediatrics are keen to move them onto adults – if they've left	Structured around AYA turning 16.	Not specified.	Frequently completed but often subpar quality.<br><br>Monash and RCH adult teams have access to paediatric medical records.	Limited referrals to transition services.	Some co-consultation / transition clinics offered.	Often provide a 'backstop' appointment while AYA waits to be seen by adult services.	

	school, they definitely get discharged from paediatrics.<br><br>Private practice continues to see AYAs up to age 19 – 22.							

Abbreviations

AYA	Adolescents and young adults
ED	Emergency department
HCP	Healthcare professional
YACDS	Young adult complex disability service
YADs	Young adults diabetes clinic

Supplementary Table 3

Standards for Reporting Qualitative Research (SRQR)*

http://www.equator-network.org/reporting-guidelines/srqr/

Page/line no(s).

Title and abstract

**Title** - Concise description of the nature and topic of the study Identifying the study as qualitative or indicating the approach (e.g., ethnography, grounded theory) or data collection methods (e.g., interview, focus group) is recommended	Page 1
**Abstract** - Summary of key elements of the study using the abstract format of the intended publication; typically includes background, purpose, methods, results, and conclusions	Pages 2

Introduction

**Problem formulation** - Description and significance of the problem/phenomenon studied; review of relevant theory and empirical work; problem statement	Pages 3-4
**Purpose or research question** - Purpose of the study and specific objectives or questions	Page 4

Methods

**Qualitative approach and research paradigm** - Qualitative approach (e.g., ethnography, grounded theory, case study, phenomenology, narrative research) and guiding theory if appropriate; identifying the research paradigm (e.g., postpositivist, constructivist/ interpretivist) is also recommended; rationale**	Pages 4, 20
**Researcher characteristics and reflexivity** - Researchers' characteristics that may influence the research, including personal attributes, qualifications/experience, relationship with participants, assumptions, and/or presuppositions; potential or actual interaction between researchers' characteristics and the research questions, approach, methods, results, and/or transferability	Page 22
**Context** - Setting/site and salient contextual factors; rationale**	Page 20-21
**Sampling strategy** - How and why research participants, documents, or events were selected; criteria for deciding when no further sampling was necessary (e.g., sampling saturation); rationale**	Page 21
**Ethical issues pertaining to human subjects** - Documentation of approval by an appropriate ethics review board and participant consent, or explanation for lack thereof; other confidentiality and data security issues	PAge 21
**Data collection methods** - Types of data collected; details of data collection procedures including (as appropriate) start and stop dates of data collection and analysis, iterative process, triangulation of sources/methods, and modification of procedures in response to evolving study findings; rationale**	PAge 22

**Data collection instruments and technologies** - Description of instruments (e.g., interview guides, questionnaires) and devices (e.g., audio recorders) used for data collection; if/how the instrument(s) changed over the course of the study	
**Units of study** - Number and relevant characteristics of participants, documents, or events included in the study; level of participation (could be reported in results)	Page 31
**Data processing** - Methods for processing data prior to and during analysis, including transcription, data entry, data management and security, verification of data integrity, data coding, and anonymization/de-identification of excerpts	Page 22
**Data analysis** - Process by which inferences, themes, etc., were identified and developed, including the researchers involved in data analysis; usually references a specific paradigm or approach; rationale**	Pages 22-23
**Techniques to enhance trustworthiness** - Techniques to enhance trustworthiness and credibility of data analysis (e.g., member checking, audit trail, triangulation); rationale**	Page 23

Results/findings

**Synthesis and interpretation** - Main findings (e.g., interpretations, inferences, and themes); might include development of a theory or model, or integration with prior research or theory	Pages 4-16
**Links to empirical data** - Evidence (e.g., quotes, field notes, text excerpts, photographs) to substantiate analytic findings	PAges 31-35

Discussion

**Integration with prior work, implications, transferability, and contribution(s) to the field -** Short summary of main findings; explanation of how findings and conclusions connect to, support, elaborate on, or challenge conclusions of earlier scholarship; discussion of scope of application/generalizability; identification of unique contribution(s) to scholarship in a discipline or field	Pages 16-19
**Limitations** - Trustworthiness and limitations of findings	Page 18

Other

**Conflicts of interest** - Potential sources of influence or perceived influence on study conduct and conclusions; how these were managed	Page 25
**Funding** - Sources of funding and other support; role of funders in data collection, interpretation, and reporting	Page 24

*The authors created the SRQR by searching the literature to identify guidelines, reporting standards, and critical appraisal criteria for qualitative research; reviewing the reference lists of retrieved sources; and contacting experts to gain feedback. The SRQR aims to improve the transparency of all aspects of qualitative research by providing clear standards for reporting qualitative research.

**The rationale should briefly discuss the justification for choosing that theory, approach, method, or technique rather than other options available, the assumptions and limitations implicit in those choices, and how those choices influence study conclusions and transferability. As appropriate, the rationale for several items might be discussed together.

Reference:
O'Brien BC, Harris IB, Beckman TJ, Reed DA, Cook DA. **Standards for reporting qualitative research: a synthesis of recommendations.** *Academic Medicine*, Vol. 89, No. 9 / Sept 2014
DOI: 10.1097/ACM.0000000000000388
